# Alternative Polyadenylation Modification Patterns Reveal Essential Posttranscription Regulatory Mechanisms of Tumorigenesis in Multiple Tumor Types

**DOI:** 10.1155/2020/6384120

**Published:** 2020-06-15

**Authors:** Min Li, XiaoYong Pan, Tao Zeng, Yu-Hang Zhang, Kaiyan Feng, Lei Chen, Tao Huang, Yu-Dong Cai

**Affiliations:** ^1^School of Life Sciences, Shanghai University, Shanghai 200444, China; ^2^Institute of Image Processing and Pattern Recognition, Shanghai Jiao Tong University, and Key Laboratory of System Control and Information Processing, Ministry of Education of China, 200240 Shanghai, China; ^3^Key Laboratory of Systems Biology, Institute of Biochemistry and Cell Biology, Chinese Academy of Sciences, Shanghai 200031, China; ^4^Shanghai Institute of Nutrition and Health, Shanghai Institutes for Biological Sciences, Chinese Academy of Sciences, Shanghai 200031, China; ^5^Department of Computer Science, Guangdong AIB Polytechnic, Guangzhou 510507, China; ^6^College of Information Engineering, Shanghai Maritime University, Shanghai 201306, China

## Abstract

Among various risk factors for the initiation and progression of cancer, alternative polyadenylation (APA) is a remarkable endogenous contributor that directly triggers the malignant phenotype of cancer cells. APA affects biological processes at a transcriptional level in various ways. As such, APA can be involved in tumorigenesis through gene expression, protein subcellular localization, or transcription splicing pattern. The APA sites and status of different cancer types may have diverse modification patterns and regulatory mechanisms on transcripts. Potential APA sites were screened by applying several machine learning algorithms on a TCGA-APA dataset. First, a powerful feature selection method, minimum redundancy maximum relevancy, was applied on the dataset, resulting in a feature list. Then, the feature list was fed into the incremental feature selection, which incorporated the support vector machine as the classification algorithm, to extract key APA features and build a classifier. The classifier can classify cancer patients into cancer types with perfect performance. The key APA-modified genes had a potential prognosis ability because of their significant power in the survival analysis of TCGA pan-cancer data.

## 1. Introduction

Cancer is one of the most threatening human diseases and ranks second to infectious diseases and cardiovascular diseases. According to statistical data provided by the World Health Organization (WHO) in 2015, cancer accounts for more than 8.8 million deaths worldwide with more than 14 million new cases and a high growth incidence [1]. Among various risk factors of cancer initiation and progression, pathogenic genetic variants and modifications, such as alternative polyadenylation (APA), are remarkable endogenous contributors, directly triggering the malignant phenotype of cancer cells [2].

APA is a specific RNA modification process contributing to gene expression regulation by generating RNA with different 3′ terminals from a single gene with multiple polyadenylation sites [3]. APA affects biological processes at a transcriptional level in various ways. First, tissue-specific APA can rapidly respond to extracellular cues, regulating the expression level of certain genes as cellular “stress” responses [4]. As evidence confirmed in pancreatic cancer, the APA of ZEB1 rapidly responds to genotoxic stress and promotes gene expression, thereby improving the adaptability of tumor cells in a flexible tumor microenvironment [5]. Second, APA may regulate different metabolisms in living cells by affecting the subcellular localization of certain protein products. APA contributes to the regulation of 3′UTR-dependent protein localization by modifying the 3′UTR, thereby affecting the widespread trafficking mechanisms for different membrane proteins, including CD47, CD44, and ITGA1 [6, 7]. Third, considering that 3′UTR is involved in multiple splicing events, APA influences posttranscriptional splicing processes and further induces the abnormal production of improper protein isoforms [8–10]. In 2014, a report on the alternative intronic polyadenylation of IL6 *trans*-signaling inhibitor confirmed that different polyadenylation patterns of the same gene (sgp130-E10) may produce different protein isoforms with different biological functions [11]. With numerous regulatory contributions to downstream biological processes, APA is also regulated by various upstream biological mechanisms involving RNA-processing factors and RNA-binding proteins, which constitute a complicated and functional interaction network for posttranscriptional regulation [12].

APA is functionally related to tumorigenesis as a key functional component of pathogen posttranscriptional regulation [13–15]. APA can be involved in tumorigenesis at three levels based on original physical functions: gene expression, protein subcellular localization, and transcription splicing patterns [4]. For example, in gene expression regulation, APA promotes the tumorigenesis of non-small-cell lung cancer by regulating the expression levels of various genes, including PABPN1, CPEB1, and E2F1, and several proliferation markers, such as MKI67, TOP2A, and MCM2 [14].

More than 30% of mRNAs have specific APA sites independent of cell types [4]. Considering that the expression profiles of different cancer types vary, we can infer that the APA sites and status of different cancer types may have diverse modification patterns and regulatory mechanisms on transcripts. Therefore, in this study, we adopted several machine learning algorithms to screen the potential APA sites at the whole genomic level in multiple tumor types and tried to find out the key APA-modified genes that might distinguish different tumor types. The TCGA-APA dataset was first analysed by the feature selection method, minimum redundancy maximum relevancy (mRMR) [16]. A feature list was obtained. Then, the incremental feature selection (IFS) [17], incorporating a support vector machine (SVM) [18], was applied on such a list to extract essential APA features. Most of the key genes corresponding to essential APA features showed a significant power in the survival analysis of TCGA pan-cancer data. Furthermore, the SVM classifier with the extracted essential APA features gave a perfect performance. This study possibly identified tumor-specific APA targets, revealed the irreplaceable role of APA modification patterns for tumorigenesis in multiple tumor types, and proposed APA sites and status as potential tumor biomarkers for the first time.

## 2. Materials and Methods

### 2.1. Datasets

The TCGA-APA dataset was downloaded from Synapse under the accession number of syn7888354 [19]. In the original dataset, 9396 APAs were obtained in 5765 patients with cancer, but several values were missing. APAs with missing values in more than 50% of samples and patients with cancer with missing values in more than 50% of APAs were removed. A total of 7544 APAs and 5709 patients were finally obtained from 17 cancer types. The remaining missing values were imputed using *K*-NN methods (*K* = 3) by R/Bioconductor package impute. The categories of 17 cancer sites and their corresponding sample sizes are listed in Table 1.

### 2.2. Feature Selection

First, mRMR [16] was conducted to rank input features, that is, APA sites, to choose a refined feature set that had better discriminatory power than the original whole set. mRMR is a widely utilized filter-based feature selection method proposed by Peng et al. [16] on the basis of two criteria: (1) relevancy between feature and category must be large and (2) redundancy between features themselves must be small [20–22]. Given a dataset with *m* features, the mRMR follows the above criteria to select features one by one and added them into a feature list, which is empty initially. In detail, for each of the remaining features, its relevance to targets (class labels) was evaluated by mutual information and its redundancies were assessed to already-selected features. The feature with maximum relevance and minimum redundancy is selected and added to the current feature list. The obtained feature list was called the mRMR feature list. The mRMR program we used was downloaded from http://home.penglab.com/proj/mRMR/index.htm. Default parameters were used to perform such a program.

Second, IFS [17] and SVM [18] were integrated to select discriminatory features and their combination. A series of feature subsets was generated on the basis of the ranked features from mRMR. Then, the classification performance of SVMs on the samples consisting of the generated feature subsets was evaluated. In the end, the feature subset with the best performance called optimum APA features, such as APA-modified genes, was selected.

SVM is a supervised learning model that can be used to analyse data, recognize feature patterns, and perform classification and regression analysis [18, 23–30]. The SVM constructs a hyperplane with a maximum margin between two groups of samples in a high-dimensional or infinite-dimensional space. SVM is also used to fit nonlinear data by mapping nonlinear data in a low-dimensional space to a high-dimensional space by a kernel trick. SVMs can also be extended for a multiclass problem by learning multiple binary SVM classifiers, and each classifier is used to classify one class from other classes. To quickly implement SVM, the tool “SMO” in Weka [31] was adopted in this study. The training procedures of this type of SVM are optimized by the sequential minimal optimization algorithm [32]. Default parameters were used. The kernel was a polynomial function, and the regularization parameter *C* was set to 1.

### 2.3. Performance Measurement

Performance measurement is an effective experimental estimation to assess the generalization performance of machine learning and can be used as an evaluation measurement to estimate the generalization performance of a learned model. In comparing different models, performance measurements should be objective and reflect the accuracy of models. Matthew's correlation coefficient (MCC) [33–38] for measuring multiclass classification performance is applied and formulated as follows:

(1) $$\begin{array}{c} \text{MCC}=\frac{\mathrm{cov}\left(X,Y\right)}{\sqrt{\mathrm{cov}\left(X,X\right)\mathrm{cov}\left(Y,Y\right)}}=\frac{\sum_{i=1}^{n}\sum_{j=1}^{C}\left(x_{ij}-{\bar{x}}_{j}\right)\left(y_{ij}-{\bar{y}}_{j}\right)}{\sqrt{\sum_{i=1}^{n}\sum_{j=1}^{C}{\left(x_{ij}-{\bar{x}}_{j}\right)}^{2}\sum_{i=1}^{n}\sum_{j=1}^{C}{\left(y_{ij}-{\bar{y}}_{j}\right)}^{2}}}, \end{array}$$

where ${\bar{x}}_{j}$ and ${\bar{y}}_{j}$ are the means of *x*_*j*_ and *y*_*j*_, respectively; *Y* is the truth label; and *X* is the predicted label. When MCC is 1, the classifier is extremely optimal. When MCC is 0, the learned classifier is not different from a random one. If MCC is −1, the classifier is the worst.

## 3. Results

**Figure 1:**
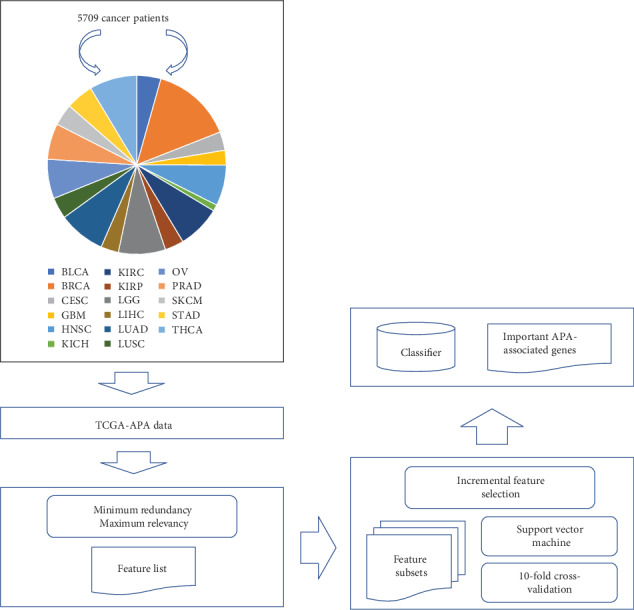
Entire procedures for the analysis of TCGA-APA data in different cancer types. The data is first analysed by the minimum redundancy maximum relevancy (mRMR) method, yielding a feature list. Then, incremental feature selection (IFS), incorporating a support vector machine, is applied on the list to extract important APA-associated genes and build an efficient classifier.

In this study, we adopted several machine learning algorithms to analyse the TCGA-APA data. The purpose was to extract essential ATA features that can correctly distinguish different cancer types. The entire procedures are illustrated in Figure 1.

### 3.1. Results of mRMR

The mRMR was first applied to the TCGA-APA data. All APA features were deeply analysed and sorted in the mRMR feature list. The obtained feature list is provided in Table S1.

### 3.2. Results of IFS with SVM

**Figure 2:**
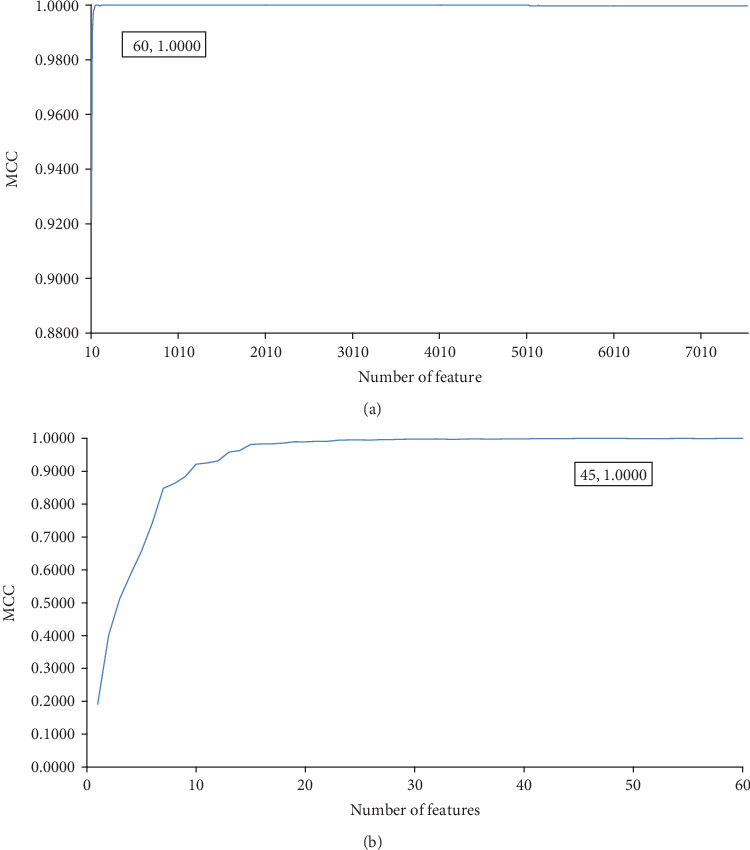
IFS curves to illustrate the performance of the support vector machine on different numbers of top features. (a) IFS curve with step 10; (b) IFS curve with 1-60 features. The perfect performance is obtained when the top 45 features are used.

Based on the mRMR feature list, the IFS method constructed feature subsets with step ten; that is, the first ten features comprised the first feature subset; then, the second feature subset further added the next ten features, and so on. On each feature subset, an SVM classifier was built with samples represented by features in the subset. 10-fold crossvalidation was conducted to evaluate the performance of each SVM classifier. The accuracy of each cancer type, overall accuracy, and MCC were counted, which are available in Table S2. To give an overview of the performance of the SVM classifier on different numbers of top features, an IFS curve is plotted in Figure 2(a), in which MCC was set as the *y*-axis and the number of features as the *x*-axis. It can be observed that the SVM classifier with lots of APA features always gave a good performance. When the top 60 features were used, the SVM classifier can provide perfect performance with MCC = 1; that is, all cancer patients were classified into the correct cancer type. To investigate whether such perfect performance can be obtained with fewer features, we constructed all possible feature subsets containing 1-60 features. Likewise, an SVM classifier was built on each of these feature subsets. Also, 10-fold crossvalidation was adopted to assess each SVM classifier. Obtained measurements are also provided in Table S2. An IFS curve was also plotted, which is shown in Figure 2(b). It can be observed that when the top 45 features were adopted, the SVM classifier also provided the perfect performance with MCC = 1. Thus, the top 45 APA features were deemed as the optimum features. Furthermore, a perfect SVM classifier was built on these features, which can be a useful tool to discriminate different tumors.

### 3.3. Results of Survival Analysis on Top Ten Features

**Figure 3:**
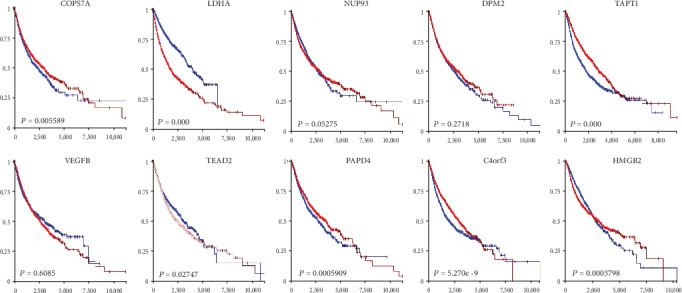
Survival analysis of the top 10 features in pan-cancer cohorts. Among the top 10 genes, seven genes had significant survival risks in the pan-cancer manner (red for the high-expression group, and blue for the low-expression group). Genes LDHA and TEAD2 are shown to predict a poor prognosis, and genes COPS7A, TAPT1, PAPD4, C4orf3, and HMGB2 are shown to indicate a good prognosis.

According to the results in Table S2, the SVM classifier with the top 10 features could reach MCC of 0.9217. This result indicated that the top ten APA features had significant APA patterns with a strong power on discriminating different tumors. These ten features are listed in Table 2. The selected APA-modified genes can discriminate different cancer types so that they would have prognostic power in a pan-cancer manner. Here, relying on the TCGA pan-cancer gene expression data and phenotype data (clinical information) [39], we firstly divided the samples into two parts according to the expression levels (expression quartiles). Using both the high-expression group and low-expression group datasets, we examined each of the top 10 genes for the survival analysis efficacy. The red survival curve shows the group of samples with a higher gene expression level, and the blue survival curve shows the group of samples with a lower expression level. In summary, the TCGA pan-cancer datasets were used to examine each of the top 10 genes based on survival analysis efficiency and are shown in Figure 3.

## 4. Discussion

### 4.1. Optimal APA-Associated Genes in Multiple Tumor Types

**Figure 4:**
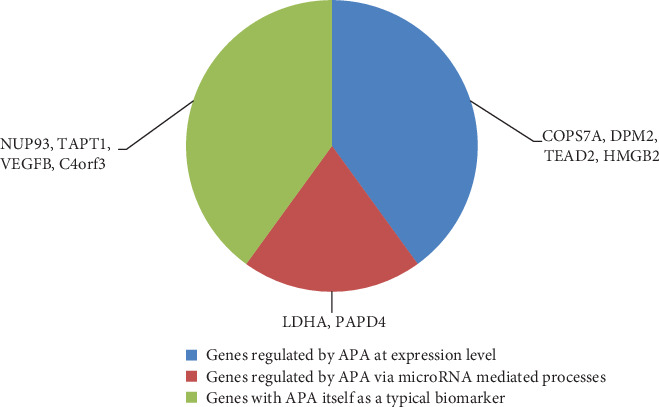
Summary of the APA-associated genes' regulatory methods on multiple tumor subtypes. Here, we summarized the three major subgroups of contributions that APA-associated genes contribute to different cancer subtypes together with the number of genes among the top 10 genes that contribute to each subgroup.

In this study, we extracted several important APA features as mentioned in Results of IFS with SVM. In addition, according to “Results of Survival Analysis on Top Ten Features,” the top ten features can really indicate different cancer types. Here, we analysed the genes related to these APA features. All these identified genes were reported and validated to have different APA patterns in most of our 17 candidate tumor types. These results validated the efficacy and accuracy of our prediction. The detailed analysis of the APA pattern of the 10 optimal genes in different candidate tumor types is presented as follows. All such 10 optimal genes have been reported to be directly related to APA during tumorigenesis. The major regulatory effects of APA on such genes have been shown at three levels (Figure 4): directly affecting the expression levels and regulating related microRNAs and APA itself as a typical biomarker.

#### 4.1.1. Genes Directly Regulated by APA at Expression Level in Multiple Tumor Types

The first examined APA-modified gene is COPS7A, which contains six potential APA sites [40]. The transcripts of COPS7A have quite different APA sites in various tissues; thus, the gene has different APA-modified patterns in various tumor types [40]. Based on mRNA sequencing data from the TCGA database, recent publications have confirmed that COPS7A has a specific expression pattern in multiple tumor types, supporting our inference from an independent aspect [41]. As a detailed case, variant bAug10 with two unique APA sites, or the transcript of COPS7A, is specifically expressed in the colon and the ovary that distinguish tumor types derived from the two tissues from other tumor types [40]. This result validated the efficacy and accuracy of our prediction.

DPM2 is another predicted gene with a unique APA status in multiple tumor types. APA regulates the specific biological function of the polyadenylation signal sequence and further contributes to the biosynthesis of dolichol phosphate-mannose in multiple mammalian cell subtypes [42]. Considering that dolichol phosphate mannose has different expression patterns in multiple cancer types, such as glioma and head and neck cancer, we can regard the APA status of DPM2 as a potential biomarker for the identification of different tumor types [43].

TEAD2, another predicted gene with an APA-modified pattern, may also have different APA modification patterns in various tumor types. A specific pattern of polyadenylation (AATAAA) on TEAD-2 regulates the expression of our predicted gene TEAD-2 involved in early mouse development, implying that this gene is regulated by APA modification [44]. In terms of the contribution of TEAD-2 to tumorigenesis, the APA modification of TEAD-2 may be functionally related to liver cancer development [45], suggesting that this gene may be a potential biomarker for the identification of a particular tumor type.

The next gene in our top-ranked prediction list is HMGB2, which is a member of the nonhistone chromosomal high-mobility group protein family. A specific study on non-small-cell lung cancer transcriptome confirmed early in 2008 that the polyadenylation pattern may directly affect the progression of lung cancer [46]. The APA modification of HMGB2 may also be involved in thyroid cancer cells [46]. Therefore, in candidate tumor types, HMGB2, an APA site-targeting gene, may be differentially expressed or regulated in many tumor types, validating the efficacy and accuracy of our prediction.

#### 4.1.2. Genes Regulated by APA via MicroRNA-Mediated Processes

LDHA is another gene with a differentially APA-modified pattern in candidate tumor types. With 14 potential APA sites, LDHA is differentially APA modified in different tissues [47]. A recent study on hepatocellular carcinoma cells has confirmed that APA-modified LDHA may directly participate in tumorigenesis by regulating the biological functions of microRNAs, validating the specific contribution of APA modification to LDHA [47]. Therefore, in the candidate tumor types, the APA pattern of LDHA may contribute to the identification of LIHC on the basis of the abovementioned evidence.

The predicted gene PAPD4 is another specific biomarker for the identification of different tumor types. In contrast to other predicted genes, PAPD4 can participate in the polyadenylation of target mRNAs, indicating its specific contribution to APA [48]. In terms of the contribution of APA to PAPD4, a study in 2014 reported that the APA modification of PAPD4 regulated by HBx may directly contribute to the HBV-related dysregulation of miR-122 [49]. Biological processes, that is, HBV-related dysregulation of miRNA, are functionally associated with hepatocellular carcinoma (LIHC), reflecting the cancer subtyping potential of PAPD4 [50].

#### 4.1.3. Genes with APA Itself as a Typical Biomarker

The next predicted gene with differential APA patterns in different tumor types is NUP93. The transcript information from the NCBI AceView database supports NUP93 with five validated APA-modified sites, confirming the potential of APA-mediated transcription regulation on this gene [40]. In our study, NUP93 was APA modified in gastrointestinal diseases, including pancreatic cancer; thus, the APA status of NUP93 may be a potential indicator for the identification of pancreatic cancer [51]. The APA status of NUP93 in glioma, in addition to pancreatic cancer, is also tumor-specific [52, 53]. Therefore, with a unique APA status in pancreatic cancer and glioma, the predicted NUP93 may be an effective indicator for the identification of different tumor types.

TAPT1 has specific APA patterns in multiple tumor types. As a transmembrane protein, the APA modification of this gene affects the stability of the encoded protein's transmembrane structure [54, 55]. In terms of the contribution of APA to TAPT1 in different tumor types, APA modification affects the 3′UTR of TAPT1 in hepatocellular carcinoma cell lines [56], indicating that this specific pattern of APA modification may contribute to the identification of LIHC from other tumor types.

VEGFB is functionally modified by APA under multiple physical and pathological conditions [57]. With five validated APA sites, the abnormal APA-modified transcripts of VEGFB contribute to the pathogenesis of chronic liver disease and even LIHC, and this finding was consistent with our prediction [58]. Similarly, the specific APA modification of VEGFB may be involved in the functional regulation of CPEB1 and CPEB4 [58], further contributing to the tumorigenesis of multiple tumor types, including cervical, ovarian, and glioma cancers [59]. Therefore, the specific pattern of APA modification on our predicted VEGFB might also be an applicable biomarker of different tumor types, validating the efficacy and accuracy of our prediction.

C4orf3 is a predicted gene that may be functionally related to APA-mediated tumorigenesis. It is APA modified and forms a functional fusion transcript named KLHL2-C4orf3 fusion transcript, which is merged by the second, third, and fourth exons of KLHL2 and the first intron of C4orf3 [60]. Therefore, the modified APA sites of C4orf3 may be a potential APA target of the fusion transcript KLHL2-C4orf3. Considering that this fusion transcript is functionally related to lung adenocarcinoma but not to other tumor types, specific APA-modified patterns of C4orf3-induced transcript or fusion transcript may be potential biomarkers for the identification of lung adenocarcinoma [60].

### 4.2. Optimal APA-Associated Genes in Pan-Cancer Survival Analysis

APA-modified genes can participate in multiple cancer development and progression. As such, APA-modified genes play important roles in pan-cancer tumorigenesis, including potential prognosis power in pan-cancer cohorts. Thus, the survival risk of each APA gene (Figure 4) was evaluated on the basis of the TCGA pan-cancer gene expression data and phenotype data [39]. For each APA gene, the pan-cancer samples were divided into two groups based on expression quartiles, where one group of the samples had a high gene expression (red survival curve) and the other group of the samples had a low gene expression (blue survival curve). Of the 10 APA genes, 7 had significant survival risks in a pan-cancer manner. LDHA and TEAD2 were oncogenes whose high expression levels indicated a poor survival expectation. By contrast, other genes, including COPS7A, TAPT1, PAPD4, C4orf3, and HMGB2, have a tumor suppressor effect, and their high expression levels characterize patients with a good survival potential. Thus, 70% of our 10 APA genes had satisfactory prognostic power in a pan-cancer way, thereby supporting our analysis efficiency.

Overall, this study examined 10 potential gene biomarkers with differential APA-modified patterns in different tumor types. The 10 identified biomarkers were validated by recent publications, reflecting the efficacy and accuracy of the study. The presented computational approach might contribute to the identification of potential APA target sites at a whole genome level and provide a new approach to reveal the significant role of APA-induced RNA modification underlying tumorigenesis.

## 5. Conclusions

This study analysed the APA sites for multiple tumor types using several computational methods. Several key APA-modified genes were extracted, which can distinguish different tumor types; that is, they can be potential tumor biomarkers.

## Figures and Tables

**Table 1:** Summary of the used dataset.

Index	Cancer type	Sample size
1	Bladder urothelial carcinoma (BLCA)	249
2	Breast invasive carcinoma (BRCA)	837
3	Cervical squamous cell carcinoma and endocervical adenocarcinoma (CESC)	191
4	Glioblastoma multiforme (GBM)	152
5	Head and neck squamous cell carcinoma (HNSC)	422
6	Kidney chromophobe (KICH)	66
7	Kidney renal clear cell carcinoma (KIRC)	446
8	Kidney renal papillary cell carcinoma (KIRP)	195
9	Brain lower-grade glioma (LGG)	486
10	Liver hepatocellular carcinoma (LIHC)	183
11	Lung adenocarcinoma (LUAD)	486
12	Lung squamous cell carcinoma (LUSC)	220
13	Ovarian serous cystadenocarcinoma (OV)	407
14	Prostate adenocarcinoma (PRAD)	370
15	Skin cutaneous melanoma (SKCM)	225
16	Stomach adenocarcinoma (STAD)	282
17	Thyroid carcinoma (THCA)	492

**Table 2:** Top 10 features selected by the mRMR method.

Feature index	Gene name	Score
1	NM_001164095|COPS7A|chr12|+	0.91171
2	NM_001165415|LDHA|chr11|+	0.73417
3	NM_001242795|NUP93|chr16|+	0.68580
4	NM_003863|DPM2|chr9|-	0.66010
5	NM_153365|TAPT1|chr4|-	0.65060
6	NM_003377|VEGFB|chr11|+	0.63976
7	NM_001256661|TEAD2|chr19|-	0.63742
8	NM_001114394|PAPD4|chr5|+	0.62491
9	NM_001001701|C4orf3|chr4|-	0.61409
10	NM_002129|HMGB2|chr4|-	0.61438

## Data Availability

Previously reported data were used to support this study and are available at Synapse. These prior studies (and datasets) are cited at relevant places within the text as references [19].

## Supplementary Materials

Supplementary 1 Table S1: ranked gene list from the mRMR method.

Supplementary 2 Table S2: classification performance yielded by the IFS method and SVM classifiers with different numbers of features.
